# Omics-Based Comparative Transcriptional Profiling of Two Contrasting Rice Genotypes during Early Infestation by Small Brown Planthopper

**DOI:** 10.3390/ijms161226128

**Published:** 2015-12-03

**Authors:** Weilin Zhang, Ling Yang, Mei Li, Bojun Ma, Chengqi Yan, Jianping Chen

**Affiliations:** 1 College of Chemistry and Life Sciences, Zhejiang Normal University, Jinhua 321004, China; wlzhangwhu@163.com (W.Z.); yangl@zjnu.edu.cn (L.Y.); mbj@zjnu.edu.cn (B.M.); 2 Analysis Center of Agrobiology and Environmental Sciences, Zhejiang University, Hangzhou 310058, China; limei7251314@zju.edu.cn; 3 State Key Laboratory Breeding Base for Zhejiang Sustainable Pest and Disease Control, Ministry of China Key Laboratory of Biotechnology in Plant Protection, Institute of Virology and Biotechnology, Zhejiang Academy of Agricultural Sciences, Hangzhou 310021, China

**Keywords:** rice, small brown planthoper, Pathway Tools Omics Viewer, defense

## Abstract

The small brown planthopper (SBPH) is one of the destructive pests of rice. Although different biochemical pathways that are involved in rice responding to planthopper infestation have been documented, it is unclear which individual metabolic pathways are responsive to planthopper infestation. In this study, an omics-based comparative transcriptional profiling of two contrasting rice genotypes, an SBPH-resistant and an SBPH-susceptible rice line, was assessed for rice individual metabolic pathways responsive to SBPH infestation. When exposed to SBPH, 166 metabolic pathways were differentially regulated; of these, more than one-third of metabolic pathways displayed similar change patterns between these two contrasting rice genotypes; the difference of change pattern between these two contrasting rice genotypes mostly lies in biosynthetic pathways and the obvious difference of change pattern lies in energy metabolism pathways. Combining the Pathway Tools Omics Viewer with the web tool Venn, 21 and 6 metabolic pathways which potentially associated with SBPH resistance and susceptibility, respectively were identified. This study presents an omics-based comparative transcriptional profiling of SBPH-resistant and SBPH-susceptible rice plants during early infestation by SBPH, which will be very informative in studying rice-insect interaction. The results will provide insight into how rice plants respond to early infestation by SBPH from the biochemical pathways perspective.

## 1. Introduction

Rice (*Oryza sativa* L.) is an important food crop and a source of calories for billions of people [1]. Many insects attack rice. Of these insects, the brown planthopper (BPH; *Nilaparvata lugens* Stål), the small brown planthopper (SBPH; *Laodelphax striatellus* Fallén) and the white-backed planthopper (WBPH; *Sogatella furcifera* Horváth) are three major planthoppers attacking rice, causing significant loss of rice yield [2]. Understanding the defense responses of rice to planthopper infestation is important for developing strategies for such insect control. Studies of the molecular responses of rice to these species of sucking insects have shown that rice responses to these species of sucking insects are regulated through a complex network of salicylic acid (SA)- and jasmonic acid (JA)/ethylene (ET)-dependent signaling pathways [3,4,5,6,7,8]. Gene expression profiles and analyses of physiological responses suggested that the genes involved in several different pathways including cell defense, cellular transport, metabolism, signal transduction, macromolecular degradation and biogenesis, cellular communication and plant defenses were found to be significantly differentially regulated by BPH/SBPH feeding [3,7,9,10,11,12]. Transcription factors (TFs) play a key role in regulation of transcriptional expression in biological processes [13]. Microarray analysis suggested that rice TFs were involved in defense response to BPH attack [8,14] and over-expression of rice TFs in transgenic rice plants increased resistance to WBPH [15]. Although gene expression profiles resulting from planthopper infestation have been studied by several groups by using two contrasting rice genotypes, the defense mechanisms of rice against these species of sucking insects remain to be largely explored.

In attempt to gain insight into the molecular mechanisms of rice resistance to SBPH, in this study, two contrasting rice genotypes, an SBPH-resistant introgression line (Pf9279-4; R) and its SBPH-susceptible parent (02428; S), were assessed for rice plant responses to SBPH attack at the molecular level. Previously, two-dimensional fluorescence difference gel electrophoresis analysis of these two contrasting rice genotypes that sampled at different time-points after SBPH infestation showed that the differential protein expression between these two contrasting rice genotypes were obvious within 6 h of infestation by SBPH. We therefore examined the comparative transcriptional profiling of these two contrasting rice genotypes which infested with SBPH for 0 h and 6 h by using Affymetrix GeneChip analysis. Although different biochemical pathways including cell defense, cellular transport, metabolism, signal transduction, macromolecular degradation and biogenesis, cellular communication and plant defenses have been documented [3,7,9,10,11,12], it is unclear which individual pathways are involved in the response to planthopper infestation in rice. The increasing number of sequenced plant genomes and advances in bioinformatics strategies offer immense opportunities to study biological processes related to biotic and/or abiotic stress at the cellular and whole-organism level by using a novel systems-level approach [16]. Among the bioinformatics strategies, because of the unique ability to display the differentially regulated individual biochemical pathways with omics data painted onto the pathway, the Pathway Tools Omics Viewer (http://pathway.iplantcollaborative.org/) was ultimately employed to visualize the expression patterns and further identify the individual pathways that are responsive to SBPH feeding in rice.

The Pathway Tools Omics Viewer, an integrated tool in the Gramene “Cyc” Pathways databases (http://www.gramene.org), provides visual analysis of whole-organism datasets and maps gene expression data sets to the metabolic pathways [17]. The ability to visualize the expression patterns facilitates the ability to pinpoint and interpret expression patterns of interest [16]. Of the Gramene “Cyc” Pathways databases, the RiceCyc pathway database now features 306 metabolic pathways; 2103 enzymatic reactions, 87 transport reactions, 1543 compounds and metabolites and 6040 protein coding genes are mapped to these reactions and pathways (http://pathway.iplantcollaborative.org/). In the present study, to understand the global gene expression profiles, the transcriptomic data was uploaded to visualize expression patterns by using the Pathway Tools Omics Viewer. To identify the SBPH resistance and susceptibility-related metabolic pathways, by using Pathway Tools Omics Viewer, the individual metabolic pathways that were differentially regulated were displayed with omics data painted onto the pathway; by combining Pathway Tools Omics Viewer with the web tool Venn (http://bioinfogp.cnb.csic.es/), 21 and 6 metabolic pathways potentially related to SBPH resistance and susceptibility in rice were identified, respectively. This study presents an omics-based comparative transcriptional profiling of SBPH-resistant and SBPH-susceptible rice plants during early infestation by SBPH and aims to identify individual pathways that are responsive to early infestation by SBPH in rice. The identified biochemical pathways will provide insight into how rice plants respond to early infestation by SBPH and the results will help us understand the general defense responses of rice plants to insect infestation from the perspective of biochemical pathways. 

## 2. Results

### 2.1. The Global Transcriptional Profiling of Rice In Response to Small Brown Planthopper (SBPH) Infestation

According to the results of our previous comparative two-dimensional fluorescence difference gel electrophoresis analysis, significant differences in protein expression level between these two contrasting rice genotypes sampled at different time-points after SBPH infestation were obvious within 6 h of infestation. To gain better understanding on how rice plants respond to SBPH attack at the molecular level, the transcriptional profiling of SBPH-resistant introgression line (Pf9279-4) and its SBPH-susceptible parent (02428) that had been fed upon by SBPH for 0 and 6 h was thusly examined via Affymetrix GeneChip analysis. To analyze the molecular mechanism underlying rice plant SBPH resistance, the expression levels of all rice genes in the resistant and susceptible rice plants that had been fed upon by SBPH for 0 and 6 h were examined and the comparison between before and after infestation was made and named as S6_S0 and R6_R0 in the resistant and susceptible rice plants, respectively. To gain an understanding of the basis of defense, comparisons of gene expression were also made between resistant and susceptible rice plants and named as R0_S0 and R6_S6 before and after infestation, respectively. The microarray experiments identified a total of 57,381 probe sets expressed in the four comparisons. In the comparison of rice plants that had been fed upon by SBPH for 0 and 6 h, a total of 2730 differentially expressed genes were identified in the resistant rice plants (R6_R0), while a total of 3024 differentially expressed genes were identified in the susceptible rice plants (S6_S0). In the resistant and susceptible comparison, a total of 2299 differentially expressed genes were identified when the rice plants had been fed upon by SBPH for 0 h (R0_S0), while a total of 2891 differentially expressed genes were identified when the rice plants had been fed upon by SBPH for 6 h (R6_S6). 

### 2.2. The Global Metabolic Map in Response to SBPH Infestation

As shown in Table S1, in the resistant and susceptible comparison, the number of the genes that appear in the overview of metabolic map is 407 and 552 in R0_S0 and R6_S6, respectively; the ratio of the genes that appear in the overview of the metabolic map to all the differentially expressed genes is about 17.7% and 19.1% in R0_S0 and R6_S6, respectively. In the comparison of rice plants that had been fed upon by SBPH for 6 and 0 h, the number of the genes that appear in the overview of metabolic map is 671 and 707 in R6_R0 and S6_S0, respectively; the ratio of the genes that appear in the overview of the metabolic map to all the differentially expressed genes is about 24.6% and 23.4% in R6_R0 and S6_S0, respectively. The cellular overview diagram was organized as follows. The functionally related pathways are grouped together, with shaded background boxes to delineate these groupings; biosynthetic pathways were positioned on the left, energy metabolism pathways in the middle and degradation pathways on the right [17]. In the overview diagram in this study, red for data values that exceed 2; purple for data values less than the inverse of 2, and black for data values in between; the 2 is the cutoff value of fold change that was employed to discriminate differential expression of genes. As shown in Table S1 (all the categories of the metabolic pathways in the cellular overview diagram are also shown in bold as follows in this paragraph), in response to SBPH infestation, **other biosynthesis** differentially altered only in the R6_R0; **respiration** differentially altered in the R6_S6 and S6_S0 as well as R0_S0; **inorganic nutrients metabolism** differentially altered in both R6_R0 and R0_S0; **C1 compounds utilization and assimilation** (utilization and assimilation of compounds containing one carbon) differentially altered in the R6_S6 and R6_R0 as well as R0_S0. In the R0_S0, in addition to **other biosynthesis**, energy metabolism pathways including **pentose phosphate pathways** and **respiration**, degradation pathways including **hormones degradation**, **secondary metabolites degradation**, **carbohydrates degradation** and **degradation/utilization/assimilation-other** were not differentially altered in all four comparisons. The remaining metabolic pathways as follows were differentially altered in all four comparisons. Biosynthetic pathways include **nucleosides and nucleotides biosynthesis**; **cofactors, prosthetic groups, electron carriers biosynthesis**; **carbohydrates** **biosynthesis**; **secondary metabolites biosynthesis**; **fatty acids and lipids biosynthesis**; **amino acids biosynthesis**; **amines and polyamines biosynthesis**; **hormones biosynthesis** and **cell structures biosynthesis**. Energy metabolism pathways include **fermentation**; **glycolysis**; **photosynthesis** and **TCA cycle** (tricarboxylicacidcycle acid cycle). Degradation pathways include **amino acids degradation**; **alcohols degradation**; **detoxification**; **carbohydrates degradation**; **fatty acid and lipids degradation** as well as **nucleosides and nucleotides degradation**.

### 2.3. An Overview of the Differentially Regulated Metabolic Pathways

The Pathway Tools Omics Viewer provides the capability to display the output as an animation for time-series experiments but not for single experiment time step. As shown in Table S1, for one comparative transcriptome analysis of interest, e.g., R6_R0, the metabolic map generated by Pathway Tools Omics Viewer is static but not animated. To analyze the molecular mechanism underlying rice plant SBPH resistance, the metabolic pathways that were differentially regulated between R6_R0 and S6_S0 were examined. To more readily visualize the difference in the differential regulation of metabolic pathways responsive to SBPH infestation between the resistant and susceptible rice plants, the cellular overview diagram of R6_R0 and S6_S0 was then taken as a combination for an animation. As viewed in the Pathway Tools Omics Viewer, where the difference exists between R6_R0 and S6_S0, the line between nodes in the cellular overview diagram will flash in red/purple (Movie S1). With the help of the animated pictures, we accurately and rapidly traced and marked the difference between R6_R0 and S6_S0 and thereafter shown that in one picture. As shown in Table S2, in response to SBPH infestation, the difference between R6_R0 and S6_S0 mostly lies in biosynthetic pathways; while the obvious difference between R6_R0 and S6_S0 lies in energy metabolism pathways; after attack by SBPH, respiration was down-regulated and glycolysis was up-regulated in the susceptible rice plants, whereas, respiration was not differentially altered and glycolysis was partially down-regulated in the resistant rice plants. As for degradation pathways, the major difference between R6_R0 and S6_S0 lies in inorganic nutrients metabolism, which was down-regulated in the resistant rice plants but was not differentially altered in the susceptible rice plants. Another difference between R6_R0 and S6_S0 lies in degradation/utilization/assimilation-other, which almost up-regulated in the resistant rice plants but was not differentially altered in the susceptible rice plants. 

To gain an understanding of the difference in the basis of defense in response to SBPH infestation, the R6_S6 and R0_S0 were also taken as the combination for an animation and marked the difference between R6_S6 and R0_S0 and then shown in one picture. As shown in Table S2, if the value of the ratio of the differentially regulated metabolic pathways between R6_S6 and R0_S0 to the all metabolic pathways contained in the cellular overview diagram is taken to discriminate whether the difference between R6_S6 and R0_S0 is significant or not, degradation pathways were the most significant, and energy metabolism pathways were the second most significant. As for biosynthetic pathways, the difference between R6_S6 and R0_S0 lies in carbohydrates biosynthesis, amino acids biosynthesis, amines and polyamines biosynthesis, fatty acids and lipids biosynthesis, hormones biosynthesis and cell structures biosynthesis. As for energy metabolism pathways, fermentation and glycolysis showed a difference between R6_S6 and R0_S0. As for degradation pathways, detoxification, C1 compounds utilization and assimilation, inorganic nutrients metabolism, hormones degradation, carbohydrates degradation, amino acids degradation, degradation/utilization/assimilation-other, fatty acid and lipids degradation displayed difference between R6_S6 and R0_S0.

### 2.4. The Global Batch Identification of Individual Metabolic Pathways by Pathway Tools Omics Viewer

To further explore the defense responses of rice plants to SBPH attack in more detail, the individual metabolic pathways were afterward screened by Pathway Tools Omics Viewer. Besides the capabilities for overlaying transcriptomic data onto the overview as a whole, the Pathway Tools Omics Viewer can also generate a table of magnified views of all pathways with omics data painted onto the pathway [17]. Therefore, we then chose the alternative display to generate a table containing all individual pathways with omics data painted onto the pathway by Pathway Tools Omics Viewer (Table S3). As shown in Supplement Table S4, in the resistant and susceptible comparison, a total of 82 and 108 metabolic pathways that associated with the differentially expressed genes were identified in R0_S0 and R6_S6, respectively. In the comparison of rice plants that had been fed upon by SBPH for 6 and 0 h, a total of 149 and 128 metabolic pathways that associated with the differentially expressed genes were determined in R6_R0 and S6_S0, respectively. Among the up-regulated metabolic pathways in response to the SBPH infestation in these two contrasting rice genotypes, 26 were shared, 13 and 11 were unique in R6_R0 and S6_S0, respectively; while the number of metabolic pathways that were down-regulated in both rice genotypes was 38, and unique in R6_R0 and S6_S0 were 23 and 5, respectively. In summary, a total of 190 differentially regulated metabolic pathways were determined in the four comparisons by using Pathway Tools Omics Viewer, accounting for nearly two third of the metabolic pathways that now features in the RiceCyc database. As shown in Table 1, in response to SBPH infestation, biosynthesis was the largest class of the metabolic pathways that were differentially regulated in both resistant and susceptible rice plants (R6_R0 and S6_S0) while degradation/utilization/assimilation was the second. Within biosynthesis grouping, secondary metabolites biosynthesis, amino acids biosynthesis and cofactors, prosthetic groups, electron carriers biosynthesis as well as carbohydrates biosynthesis were the largest subclass of the metabolic pathways that were differentially regulated. Within degradation/utilization/assimilation grouping, amino acids degradation and carbohydrates degradation were the largest subclass of the metabolic pathways that were differentially regulated. As for the R6_S6 and R0_S0, the largest class and its subclass of the metabolic pathways that were differentially regulated were the same as that of R6_R0 and S6_S0.

**Table 1:** Number of the differentially regulated metabolic pathways in response to SBPH attack.

Category	R6_R0	S6_S0	R6_S6	R0_S0
**Activation/Inactivation/Interconversion**				
Inactivation	2		2	
activation	1			
**Biosynthesis**				
Secondary metabolites biosynthesis	30	20	18	16
Amino acids biosynthesis	20	19	10	10
Cofactors, prosthetic groups, electron carriers biosynthesis	14	16	15	9
Carbohydrates biosynthesis	13	13	10	6
Nucleosides and nucleotides biosynthesis	9	9	5	5
Cell Structures biosynthesis	6	6	5	4
Fatty acids and lipids biosynthesis	6	8	7	7
Hormones biosynthesis	5	6	4	5
Amines and polyamines biosynthesis	4	2	2	2
Aromatic compounds biosynthesis	2	1	2	2
Aminoacyl-tRNA charging	1	1	1	1
**Degradation/Utilization/Assimilation**				
Amino acids degradation	14	16	15	8
Carbohydrates degradation	10	9	10	6
Inorganic nutrients metabolism	5	1		1
Fatty acid and lipids degradation	4	2	4	4
Detoxification	3	3	1	2
C1 compounds utilization and assimilation	3	2	2	1
Secondary metabolites degradation	3	2	2	1
Alcohols degradation	2	3		
Nucleosides and nucleotides degradation	2	1	1	2
Hormones degradation	1	1	1	
Polymeric compounds degradation	1	1	1	
Amines and polyamines degradation	1	1		
Aldehyde degradation	1	1		
Degradation/Utilization/Assimilation-Other	1			
Aromatic compounds degradation	1			
Nicotine degradation	1			
**Generation of Precursor Metabolites and Energy**				
Fermentation	4	3	3	3
Glycolysis	2	2	1	
Pentose phosphate pathways	2	2	1	
Photosynthesis	2	2	1	
Respiration	1	1	2	
TCA cycle	1	1	2	2
Acetyl-CoA biosynthesis			1	
Methanogenesis			1	

C1 compounds utilization and assimilation, utilization and assimilation of compounds containing one carbon; TCA cycle, tricarboxylicacidcycle acid cycle.

### 2.5. The Metabolic Pathways Potentially Related to SBPH Resistance in Rice

With the help of the Pathway Tools Omics Viewer, the interpretation of large-scale biological data sets can be analyzed and derived from transcriptome profiling experiments. For example, hypotheses about how the phenotypic changes which resulted from different kinds of factors such as stresses based on metabolic explanations can be generated [16,18]. Meanwhile, another web tool Venn (http://bioinfogp.cnb.csic.es/), can calculate the intersection(s) of lists of elements and generate an output displaying which elements are in each intersection or are unique to a certain list [19]. Therefore, based on the analysis by the Pathway Tools Omics Viewer, the web tool Venn was next employed to identify the metabolic pathways potentially related to SBPH resistance. Generally, plant defenses are classified as induced if gene expression changes after herbivore infestation [20,21,22]. Similarly, the metabolic pathways potentially related to SBPH resistance in rice were then examined in the light of the following classification:

Class I: in response to SBPH infestation, the metabolic pathway was differentially regulated in the resistant rice plants (R6_R0) but not in the susceptible rice plants (S6_S0); meanwhile, the basis of defense showed difference between resistant and susceptible rice plants after attack by SBPH (R6_S6) but not before attack by SBPH (R0_S0). 

Class II: in response to SBPH infestation, the metabolic pathway was differentially regulated in the resistant rice plants (R6_R0) but not in the susceptible rice plants (S6_S0); meanwhile, the basis of defense showed difference between resistant and susceptible rice plants before attack by SBPH (R0_S0) and after attack by SBPH (R6_S6).

Class III: in response to SBPH infestation, the metabolic pathway was differentially regulated in both resistant rice plants (R6_R0) and susceptible rice plants (S6_S0) and showed opposing differential changes in S6_S0 and R6_R0; meanwhile, the basis of defense showed difference between resistant and susceptible rice plants after attack by SBPH (R6_S6) and or not before attack by SBPH (R0_S0).

**Figure 1:**
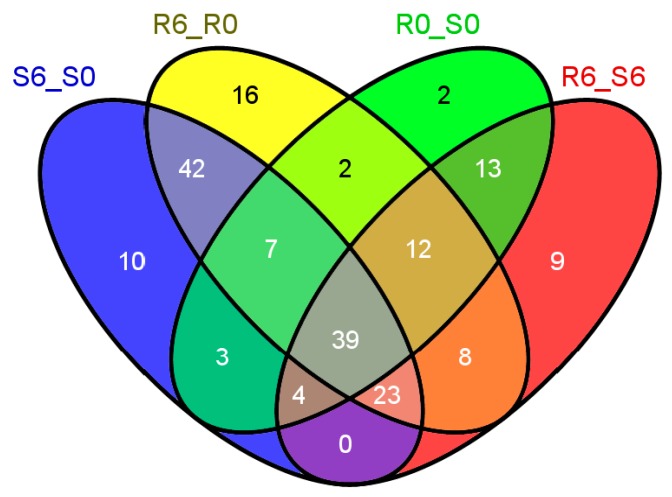
The Venn diagram showing the differentially regulated metabolic pathways identified by the Omics Viewer. The numbers in the Venn diagram are the number of the differentially regulated metabolic pathway that belongs to each category in the Venn diagram.

As shown in Figure 1 and Table S3, there were 190 metabolic pathways in the 15 categories of the Venn diagram. The number of each category was shown in the Venn diagram. For example, the number of metabolic pathway that was unique in S6_S0, R6_R0, R0_S0 and R6_S6 was 10, 16, 2 and 9, respectively. The individual metabolic pathway of each category in the Venn diagram was shown in Table S3. According to the classification of the metabolic pathways potentially related to SBPH resistance in rice, as shown in Table 2, a total of 8, 11 and 2 metabolic pathways were identified in Classes I to III, respectively (Click the individual metabolic pathway in Supplementary Tables where the individual metabolic pathway mentioned can navigate the individual metabolic pathway webpage).

**Table 2:** The identified SBPH resistance-related metabolic pathways and its differential regulation in the four comparisons.

Pathway	S6_S0	R6_R0	R0_S0	R6_S6
Class I				
Betanidin degradation		down		lower
Cytokinins degradation		down		lower
Glutamate degradation III		down		lower
IAA conjugate biosynthesis I		up		higher
IAA conjugate biosynthesis II		up		higher
Spermine biosynthesis		up		higher
Very long chain fatty acid biosynthesis		up		higher
Momilactone biosynthesis		up		higher
Class II				
Flavonoid biosynthesis		down	higher	higher
Mixed acid fermentation		down	higher	higher
Pinobanksin biosynthesis		down	higher	higher
Aminopropanol biosynthesis		down	lower	lower
Salicylate biosynthesis		down	lower	lower
Serine biosynthesis		down	lower	lower
Threonine degradation II		down	lower	lower
Threonine degradation III (to methylglyoxal)		down	lower	lower
Reductive TCA cycle I		up	higher	higher
13-LOX and 13-HPL pathway		up	lower	lower
Divinyl ether biosynthesis II (13-LOX)		up	lower	lower
Class III				
Ureide biosynthesis	up	down	lower	lower
phenylalanine degradation III	up	down		lower

IAA, indole-3-acetic acid; 13-LOX, 13-lipoxygenas; 13-HPL, 13-hydroperoxide lyase; TCA cycle, tricarboxylicacidcycle acid cycle.

### 2.6. Construction the Network of the Metabolic Pathways Potentially Related to SBPH Resistance in the SBPH-Resistant Rice Plant

**Figure 2:**
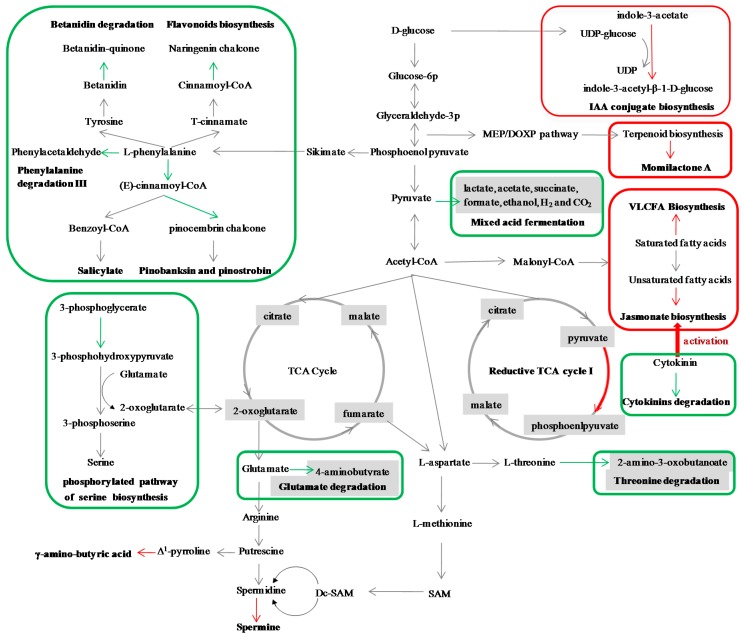
The network of the metabolic pathways potentially related to SBPH resistance in the SBPH-resistant rice plant. The up-regulated metabolic pathway was marked with red box and the red arrows denote the steps in the up-regulated metabolic pathways; the down-regulated metabolic pathway was marked with green box and the green arrows denote the steps in the down-regulated metabolic pathways. Abreviations: IAA, indole-3-acetic acid; SAM, *S*-adenosyl-methionine; Dc-SAM, decarboxylated S-adenosylmethionine; VLCFA, the very long chain fatty acid; TCA cycle, tricarboxylicacidcycle acid cycle.

Based on the classification hierarchy for individual metabolic pathways from the rice pathway database (http://pathway.iplantcollaborative.org/), the network of the metabolic pathways potentially related to SBPH resistance in the SBPH-resistant rice plant was subsequently constructed. As shown in Table 2, Table S5 and Figure 2, the primary metabolic pathways related to fatty acid (very long chain fatty acid) and amino acid (glutamate, serine, threonine and phenylalanine) and the secondary metabolic pathways related to phytoalexin (momilactone A); amine and polyamine (ureide and spermine); alkaloid (betanidin); hormone (jasmonic acid, salicylate, cytokinin and auxin); phenylpropanoid derivatives (flavonoid and pinobanksin) had been exploited potentially related to SBPH resistance in rice. The C1 compounds utilization and assimilation (reductive TCA cycle I) and fermentation (mixed acid fermentation) had also been exploited to be involved in resistance to SBPH. 

### 2.7. The Metabolic Pathways Potentially Related to SBPH Susceptibility in Rice

As in the identification of the metabolic pathways potentially related to SBPH resistance in rice, the metabolic pathways potentially related to SBPH susceptibility in rice were examined in light of the following classification:

Class I: in response to SBPH infestation, the metabolic pathway was differentially regulated in the susceptible rice plants (S6_S0) but not in the resistant rice plants (R6_R0); meanwhile, the basis of defense showed difference between resistant and susceptible rice plants after attack by SBPH (R6_S6) but not before attack by SBPH (R0_S0). 

Class II: in response to SBPH infestation, the metabolic pathway was differentially regulated in the susceptible rice plants (S6_S0) but not in the resistant rice plants (R6_R0); meanwhile, the basis of defense showed a difference between resistant and susceptible rice plants before attack by SBPH (R0_S0) and after attack by SBPH (R6_S6).

Class III: in response to SBPH infestation, the metabolic pathway was differentially regulated in both susceptible rice plants (S6_S0) and resistant rice plants (R6_R0) and showed opposing differential changes in S6_S0 and R6_R0; meanwhile, the basis of defense showed a difference between susceptible and resistant rice plants after attack by SBPH (R6_S6) and or not before attack by SBPH (R0_S0). According to the classification of the metabolic pathways potentially related to SBPH susceptibility in rice, as shown in Table 3, a total of four and two metabolic pathways were identified in Classes II to III, respectively. The phenylalanine degradation III and ureide biosynthesis that were the metabolic pathways potentially related to SBPH susceptibility in rice were the same as that of the metabolic pathways potentially related to SBPH resistance in rice. As shown in Table 3 and Table S5, the primary metabolic pathways related to amino acid (lysine degradation I, phenylalanine degradation III) and fatty acids and lipids biosynthesis (phospholipid biosynthesis II) and the secondary metabolic pathways related to cofactors, prosthetic groups, electron carriers biosynthesis (methylerythritol phosphate pathway, thiamine biosynthesis ) as well as amine and polyamine (ureide biosynthesis) had been utilized potentially related to SBPH susceptibility in rice.

**Table 3:** The identified SBPH susceptibility-related metabolic pathways and its differential regulation in the four comparisons.

Pathway	S6_S0	R6_R0	R0_S0	R6_S6
**Class II**				
methylerythritol phosphate pathway	down		lower	lower
thiamine biosynthesis	down		lower	lower
lysine degradation I	up		higher	higher
phospholipid biosynthesis II	up		lower	lower
**Class III**				
phenylalanine degradation III	up	down		lower
ureide biosynthesis	up	down	lower	lower

### 2.8. The Changes of Free Amino Acid Levels in Response to SBPH Infestation

The result of the identification of metabolic pathways potentially related to SBPH resistance and susceptibility in rice showed that several kinds of amino acids were found to be correlated with SBPH resistance or susceptibility in rice, therefore, the free amino acid levels were measured. As shown in the Table 4, in response to SBPH infestation, total free amino acid levels decreased 47% in the susceptible rice plants but there was no change in the resistant rice plants; glutamate was the most abundant amino acids and its level increased in the resistant rice plants but its level decreased in the susceptible rice plants; isoleucine level decreased greatly in the susceptible rice plants while cystathionine level decreased greatly in the resistant rice plants. The γ-amino-butyric acid showed the largest change in concentration in the resistant rice plants in response to SBPH infestation (an increase of about 900%) and the tyrosine was the second (an increase of about 300%). The tyrosine showed the largest change in concentration in the susceptible rice plants in response to SBPH infestation (an increase of about 140%). The level of arginine, phenylalanine, leucine, alanine, glycine, serine, alanine, threonine and leucine decreased in the susceptible rice plants but increased in the resistant rice plants; the level of a-amino-butyric acid decreased in the resistant plant but increased in the susceptible rice plants; the level of lysine, aspartic acid, valine, citrulline, methionine, cystine, ethanolamine, ß-aminoisobutyric acid, cystathionine and ornithine decreased in both resistant and susceptible rice plants; in addition to the level of tyrosine and γ-amino-butyric acid, the level of a-aminoadipic acid and proline also increased in both resistant and susceptible rice plants.

**Table 4:** The concentrations of free amino acids in the resistant (R) and susceptible (S) rice plants before (0 h) and after (6 h) attack by SBPH.

Free Amino Acid	Concentration of Free Amino Acids (µg per g FW) ^x^				Percent Change of Concentration	
	S0	R0	S6	R6	S6_S0	R6_R0
Isoleucine	27.7 ± 2.7	43.5 ± 4.2	1.9 ± 0.4	12.5 ± 1.4	−93.1	−71.3
Citrulline	12 ± 1.3	13 ± 1.4	2.4 ± 0.3	7 ± 0.8	−80.0	−46.2
Lysine	27 ± 2.8	31.1 ± 3.4	6.3 ± 0.6	9.8 ± 0.9	−76.7	−68.5
Methionine	11.80 ± 0.59	3.60 ± 0.28	11.03 ± 0.67	0.42 ± 0.04	−69.5	−96.2
Cystine	11.44 ± 0.99	5.97 ± 0.45	24.47 ± 1.79	10.14 ± 0.91	−47.8	−58.6
Valine	23.4 ± 1.9	23.1 ± 2.5	13.6 ± 1.5	16.8 ± 1.3	−41.9	−27.3
Aspartic acid	26.95 ± 3.59	15.68 ± 1.23	23.70 ± 2.25	21.65 ± 1.94	−41.8	−8.6
Cystathionine	2.73 ± 0.25	1.74 ± 0.13	4.58 ± 0.68	0.39 ± 0.03	−36.3	−91.5
Ornithine	2.1 ± 0.1	3.6 ± 0.1	1.4 ± 0.1	2.9 ± 0.2	−33.3	−19.4
β-Aminoisobutyric Acid	3.6 ± 0.3	5 ± 0.4	2.7 ± 0.2	1.8 ± 0.2	−25.0	−64.0
Ethanolamine	11 ± 1.3	9.1 ± 1	8.7 ± 0.9	7.5 ± 0.8	−20.9	−17.6
Phenylalanine	18.5 ± 1.8	7.5 ± 0.3	3.2 ± 0.3	7.9 ± 0.5	−82.7	5.3
Arginine	90.4 ± 10.9	30 ± 3.4	18.6 ± 1.8	46.7 ± 4.8	−79.4	55.7
β-Alanine	6.4 ± 0.3	5.5 ± 0.4	3.9 ± 0.4	8.1 ± 0.6	−39.1	47.3
Serine	7.19 ± 0.71	4.43 ± 0.31	2.87 ± 0.26	5.46 ± 0.45	−38.4	90.2
Glycine	7.7 ± 1	9.3 ± 1.6	5.2 ± 0.4	11.1 ± 1.2	−32.5	19.4
Leucine	2.2 ± 0.1	6.3 ± 0.2	1.5 ± 0.1	11.3 ± 1.3	−31.8	79.4
Glutamic Acid	114.3 ± 8.8	143.8 ± 14.2	81.9 ± 8.9	160.4 ± 15.4	−28.3	11.5
Threonine	2.9 ± 0.3	1.9 ± 0.2	2.1 ± 0.2	3.6 ± 0.3	−27.6	89.5
Alanine	10 ± 1.1	10.9 ± 1.2	8.9 ± 0.4	20 ± 0.8	−11.0	83.5
α-Amino-*n*-butyric Acid	2.5 ± 0.2	3.6 ± 0.2	4 ± 0.2	2.9 ± 0.2	60.0	−19.4
Tyrosine	4.7 ± 0.3	3.7 ± 0.3	11.3 ± 1.2	14.7 ± 1.2	140.4	297.3
α-Aminoadipic Acid	7.9 ± 0.6	11.5 ± 1.4	14.3 ± 0.8	28.4 ± 1.2	81.0	147.0
γ-Amino-n-butyric Acid	4.4 ± 0.4	2.3 ± 0.2	6.2 ± 0.5	22.9 ± 2	40.9	895.7
Proline	5 ± 0.3	2.6 ± 0.2	5.7 ± 0.3	5.3 ± 0.4	14.0	103.8
Total	443.7 ± 40.7	434.1 ± 40.9	235.3 ± 21.1	439.6 ± 37.9	−47.0	1.3

^x^ Results are mean values of three replicates ± standard error. FW means fresh weight.

## 3. Discussion

In this study, we visualized the rice transcriptomic data of microarray expression profiling by using Pathway Tools Omics Viewer. By using the capabilities of Pathway Tools Omics Viewer for overlaying transcriptomic data onto the overview as a whole and animation of the comparative transcriptome analysis, our data suggested that the difference of change pattern between these two contrasting rice genotypes mostly lies in biosynthetic pathways when exposed to SBPH; while the obvious difference of change pattern between these two contrasting rice genotypes lies in energy metabolism pathways (Table S2). By using the capabilities of Pathway Tools Omics Viewer for generation a table containing all individual pathways with omics data painted onto the pathway, 166 metabolic pathways that were differentially regulated when exposed to SBPH were identified; of these, 64 metabolic pathways displayed similar change pattern in both two contrasting rice genotypes when exposed to SBPH (Table S4); in response to SBPH infestation, biosynthesis was the largest class of the differentially regulated metabolic pathways in both resistant and susceptible rice plants (R6_R0 and S6_S0; Table 1). By combination Pathway Tools Omics Viewer and the web tool Venn, 21 and 6 metabolic pathways were considered to be potentially associated with SBPH resistance and susceptibility in rice; these 21 SBPH resistance-related metabolic pathways were related to cuticle, phytoalexin, amine and polyamine, alkaloid, hormone, amino acid, fermentation and C1 compounds utilization and assimilation; in response to SBPH infestation, the metabolic pathways derived from phenylalanine including betanidin degradation, flavonoid biosynthesis, pinobanksin biosynthesis, salicylate biosynthesis and phenylalanine degradation III were down-regulated in the resistant rice plants (Figure 2); these six SBPH susceptibility-related metabolic pathways were related to amino acid, lipid, cofactors, prosthetic groups, electron carriers biosynthesis as well as amine and polyamine. Of the metabolic pathways that were considered to be potentially associated with SBPH resistance and susceptibility in rice, the change pattern of the phenylalanine degradation III and ureide biosynthesis in the resistant rice plants displayed opposing differential changes in the susceptible rice plants (Table 2 and Table 3), indicating these two metabolic pathways are highly potentially responsive to SBPH infestation.

The plant epidermis is considered to have an important role in defense against insect attack [23]. Observations implicated that rice plant epidermis plays a role in selection of food plant by BPH and that a high ratio of long to short carbon-chain compounds in BPH resistant rice varieties largely determined planthopper feeding responses [24]. These observations provide support for the viewpoint that increased activity in response to chemical characteristics of epidermis wax may play an important role in field resistance [24]. In this study, the epidermis wax related metabolic pathway, the very long chain fatty acid biosynthesis, was found to be up-regulated in the SBPH-resistant rice plants. The very long chain fatty acids are fatty acids of 20 to 36 carbons and are required for plant cuticle biosynthesis [25,26]. Recently, the very long chain fatty acid biosynthesis pathway has been found to be associated with plant defense; well-organized cuticle layers constitute the outermost layer of epidermal cells and thereafter act as the first natural barrier when encountered by pathogens [27]. Similarly, the very long chain fatty acid biosynthesis in the SBPH resistant rice plants was up-regulated when exposed to SBPH infestation. 

It is reported that some phloem feeding insects induce the SA pathway while suppressing the plant’s JA response [28]. In response to planthopper attack, these antagonistic phenomena of SA and JA have also been observed. However, these antagonistic phenomena associated with rice resistance to planthopper seem to be in a contrasting manner. In response to BPH infestation, JA activity was suppressed while levels of SA were much higher accumulated [28]. In contrast, in this study, in the SBPH resistant rice plants, the JA biosynthesis was up-regulated while the SA biosynthesis and its branch pinobanksin biosynthesis were down-regulated. Growing evidence shows that plant defense against their different kinds of attackers is also in a hormones-dependent manner [29]. Besides JA and SA, other plant hormones such as auxin and cytokinin (CK) also serve as modulators of the hormone signaling backbone [29]. Auxin signaling is critical in regulation plant growth and development [30]. SBPH infestation seems to significantly modify auxin metabolism to decrease plant growth capacity. In the SBPH resistant rice plants, the inactivation of IAA (IAA, indole-3-acetic acid; IAA conjugate biosynthesis I and II), showed the strongest response among the 21 metabolic pathways potentially related to SBPH resistance in rice and was significantly up-regulated after attack by SBPH (Table S5). In rice-WBPH interactions, genes involved in auxin signaling such as auxin-responsive protein, auxin efflux carrier component 4, and auxin-responsive protein IAA14 were also strongly suppressed [31]. It has been reported that CK mediates insect resistance and also has an important role in the activation of JA biosynthesis [32]. For example, after wounding, the level of CK increased in transgenic tobacco (*Nicotiana tabacum* cv. Xanthinc) plants over-expressing a small GTP-binding protein, which resulted in increased rates of JA production [33]. In this study, we also observed that CK degradation was down-regulated and JA biosynthesis was up-regulated in the SBPH-resistant plant.

Betanidin is synthesized from tyrosine and is water-soluble nitrogen-containing pigment [34]. Pigment accumulation has been reported to be induced in response to insect feeding and the corresponding biosynthetic genes maybe directed against herbivores [35]. In the SBPH-resistant rice plants, betanidin degradation was down-regulated. It is therefore significant that tyrosine, a precursor of betanidin, showed the second most significant change in the SBPH-resistant rice plants, increasing about 300%. Phytoalexins are antimicrobial specialised compounds which are produced by plants as a response to pathogen attack [36]. Momilactones from rice are recognized as the major diterpenoid phytoalexins and growth inhibitors [37,38]. It has been reported that WBPH attack resulted in increased levels of momilactone A within 24 h [39]. Of the 21 metabolic pathways potentially related to SBPH resistance in rice, momilactone biosynthesis was the second most significantly altered pathway after attack by SBPH and was up-regulated in the SBPH-resistant rice plants (Table S5). In plants, flavonoids are thought to have many functions including attracting pollinating insects [40] and have been reported to stimulate the probing of rice WBPH and SBPH [41,42]. In accordance with the resistance, in the SBPH-resistant rice plants, flavonoid biosynthesis was down-regulated. 

Amino acids and amino acid derivatives have been considered to have an important role in defense response [43]. Glutamate was the most abundant amino acid in the resistant rice plants before and after attack by SBPH (Table 4). Arginine is synthesized from glutamate and is the precursor of spermine and γ-amino-butyric acid [44,45]. The level of glutamate and arginine increased in the resistant rice plants but decreased in the susceptible rice plants after attack by SBPH. The γ-amino-butyric acid was the amino acid showing the largest change in concentration in the resistant rice plants in response to SBPH infestation (an increase of about 900%). Several lines of evidence indicate that increased γ-amino-butyric acid level is related to plant defense [46]. Spermine has also been implicated in plant defenses and its intermediate putrescine has been reported to directly affect insect growth and development and therefore could also act as a defense mechanism against some herbivorous insects [47]. Thus, the coordinated and linked changes pattern of glutamate and arginine level maybe enhance spermine and γ-amino-butyric acid biosynthesis and act as part of the defense against SBPH infestation. Meanwhile, before attack by SBPH, the level of isoleucine was the second highest, whereas, after attack by SBPH, the second highest level of amino acid was replaced by arginine. The two most abundant amino acid accounts for nearly one half of the total amino acid content when exposed to SBPH. Furthermore, although the functional significance of the phosphorylated pathway of serine biosynthesis in plants is not yet known, it is clear that in the phosphorylated pathway of serine biosynthesisthe glutamate functions as the substrate for the transfer of an amino group from glutamate to 3-phosphoserine [48]; in the resistant rice plants, the phosphorylated pathway of serine biosynthesiswas down-regulated in the resistant rice plants, meaning less glutamate used in the phosphorylated pathway of serine biosynthesis. In addition, the glutamate degradation III was down-regulated in the resistant rice plants. In the last step of synthesis of isoleucine, an amino group from glutamate was transferred to produce the isoleucine (http://pathway.iplantcollaborative.org); in the resistant rice plants, isoleucine level which was the second most abundant before attack showed the third significant decreased after attack, meaning less glutamate used in synthesis of isoleucine. Collectively, the increased N-rich glutamate and arginine in the resistant rice plants means that the N-rich soluble molecules accumulated. The accumulated N-rich soluble molecules have been considered as a means of nitrogen (N) and the carbon (C) store after wounding, which helps to prevent N losses during the wounding response and could work as a source for new growth after the wound recovery phase [49,50]. Additionally, C store was also modulated in the resistant rice plants during the SBPH response by down-regulation of the mixed acid fermentation in which the products are mixed acids, particularly equal amounts of carbon dioxide (CO_2_) [51,52] and by up-regulation of the reductive TCA cycle I in which two molecules of CO_2_ can be fixed [53].

Threonine has been conferred as the amino acid that resistant to *Hyaloperonospora. arabidopsidis*, presumably by altering the pathogen’s ability to grow under that condition; mutations in threonine biosynthesis gene displayed increased resistance to *H. arabidopsidis* and increased accumulation of threonine [54]. Similarly, the threonine level increased and its expression (aminopropanol biosynthesis, threonine degradation II and threonine degradation III (to methylglyoxal)) was down-regulated in the resistant rice plants when exposed to SBPH. Hopperburn partially results from the amides accumulation in infested rice leaf blade; the amides accumulate in the leaf-blade tissues because translocation systems of amides are not functioning [55]. There are two major forms of organic nitrogen compounds that can be transported amides (such as asparagine and glutamine) and ureides (such as allantoin and allantoate) [56]. In response to SBPH infestation, the ureide biosynthesis was down-regulated in the resistant rice plants but up-regulated in the susceptible rice plants, presumably indicating the amide transportation systems are not functioning in the susceptible rice plants. 

Overall, more than one third of the 166 differentially regulated metabolic pathways displayed similar change pattern in both two contrasting rice genotypes when exposed to SBPH. Twenty-one differentially regulated metabolic pathways were associated with SBPH resistance in rice. Briefly, SBPH infestation modulated phytohormones such as SA, JA, auxin and cytokinin; particularly, SA and JA showed antagonistic phenomena; inactivation of auxin enhanced significantly and showed the strongest response, meaning a shift from growth and development to defense. Following SBPH infestation, the major diterpenoid phytoalexin momilactone A metabolism enhanced significantly and showed the second strongest response; the N-rich glutamate and arginine levels increased and were the most abundant in resistant rice plants, together with the down-regulation of mixed acid fermentation and up-regulation of the C1 compounds utilization and assimilation, suggesting a N and C store to prevent N and C losses and work as a source for new growth after the phase of wound recovery. In summary, SBPH infestation caused changes in the primary and secondary metabolism; rice plants protect themselves against SBPH at several levels. Of these, one is the well-organized cuticle layers as first barrier encountered by SBPH. The second is the biosynthesis of inducible chemical defences such as the major diterpenoid phytoalexin momilactone A. The third is a shift from growth and development to defense, enhancing the capacity of rice plants to main high fitness in the face of enemies. The fourth is the modulation of N and C store and work as a source for new growth after the phase of wound recovery. In infested resistant rice plants, metabolic processes presumably not only protect them against SBPH but also enhance the capacity of rice plants to repair the tissue that injured by SBPH attack.

## 4. Materials and Methods

### 4.1. Plant and Insect Materials

Two rice genotypes, Pf9279-4 and *O*. *sativa* (cv. 02428), the SBPH-resistant and SBPH-susceptible rice line, respectively, were selected for this study. Pf9279-4 is an introgression line derived from the asymmetric somatic hybridization between *Oryza* *officinalis* and *O*. *sativa* (cv. 02428). Pf9279-4 bears a major QTL *Qsbph3d* and two minor QTLs *Qsbph7a* and *Qsbph12b* [57]. The SBPH insects used for infestation were collected from rice fields in Jiangsu Province, China, that was confirmed to be non-viruliferous or viruliferous by dotimmunobinding assay and PCR detection [58]. The non-viruliferous population and viruliferous SBPH population were independently maintained on 02428 plants in the insect-proof in greenhouse in Zhejiang Academy of Agricultural Sciences. For simulation the natural reaction of rice attacked by SBPH in open field in this study, second to fourth instar nymphae of the mixture of the non-viruliferous and viruliferous SBPH population were transferred from 02428 and employed for infestation experiments.

### 4.2. SBPH Infestation Experiments

The resistant and susceptible rice plants were planted in pots (50 cm in length and 30 cm in width). When the seedlings were at the fourth leaf stage, weak plants were removed from the pots. In the insect-proof in greenhouse, second to fourth instar nymphs of the mixture of non-viruliferous and viruliferous SBPH population were introduced at a density of ten insects per seedling [57]. At each selected time-point, the two outmost layers of leaf sheaths were separated from the rice plants which had been fed and not fed by SBPH infestation, frozen immediately in liquid nitrogen and stored at −80 °C. The rice plants that had not been fed upon by SBPH were performed as a control experiment and its two outmost layers of leaf sheaths were separated and collected as controls.

### 4.3. Affymetrix GeneChip Analysis

According to the results of our previously comparative two-dimensional fluorescence difference gel electrophoresis analysis, significant differences in protein expression level between these two contrasting rice genotypes that sampled at different times after SBPH infestation were obvious within 6 h of infestation. Therefore, the two outmost layers of leaf sheaths of these two contrasting rice genotypes that had been fed upon by SBPH for 6 h and controls were used for microarray experiments. Beijing Biochip Co., Ltd. carried out the microarray experiments and data analysis. RNA purification, cDNA probe preparation, target hybridization, washing, staining, scanning, image analysis and the initial data processing were performed according to Affymetrix GeneChip^®^ Expression Analysis Technical Manual. Three biological replications were performed. Expression levels were compared with those in Affymetrix^®^ Microarray Suite version 5.0 (MAS 5.0, Sacramento, CA, USA). Significantly differentially expressed genes were screened in the Significant Analysis of Microarray software (SAM, version 3.02, Palo Alto, CA, USA) [59]. Significantly differentially expressed genes were determined with a fold change of  ≥2.0 and a *q*-value of <0.05 in the SAM output result. Checks of annotations from Affymetrix for significant differentially expressed genes were performed by using Blastx program in NCBI public database [60] and performed in TIGR Rice Genome Annotation version 4.0 (http://rice.tigr.org) [61]; corrected if necessary.

### 4.4. Validating of Rice Transcriptome Data for Pathway Tools Omics Viewer

The Pathway Tools Omics Viewer maps the expression values is in Gene ID-dependent manner [17]. Therefore, batch translation all probe set ids of the all differentially expressed genes to corresponding TIGR locus ids by using the Gene List Suite tool at the Plant Expression Database (http://www.plexdb.org) or by using the tool Omics Validator at the Gramene “Cyc” Pathways databases (http://pathway.iplantcollaborative.org/). For single experiment time step, listing all differentially expressed gene in a *microsoft* excel worksheet. The table in the *microsoft* excel worksheet contains 2 columns, the first row contains column header following TIGR locus id and the relative or absolute expression level of each differentially expressed gene, one gene per row. In the Pathway Tools Omics Viewer, for column numbering purposes, the first column, which contains the TIGR locus id, is column number 0 and the first potential data column is column number 1 [17]. Therefore, for time series, listing all differentially expressed gene in a *microsoft* excel worksheet, the first row contains column header following TIGR locus id and column numbers (with each column number corresponding to the relative or absolute expression level of each differentially expressed gene for a single timepoint), one gene per row. The microarray expression data is then saved as a tab-delimited data file and stored on the local computer [17]. 

### 4.5. Visualization of Rice Transcriptome Data Using Pathway Tools Omics Viewer

This section was performed as follows according to the guidelines at Pathway Tools Omics Viewer webpage (http://pathway.iplantcollaborative.org). It is worth noting that the rice pathway database should be first selected when the rice transcriptomic data was visualized [17]. Open the Pathway Tools Omics Viewer webpage (http://pathway.iplantcollaborative.org), click on the “change” link. In the dialog that pops up, select the Oryza sativa Japonica Group, then click OK to exit the dialog. The Pathway Tools Omics Viewer is next selected. Upload the transcriptomic data that validated as noted above. In the column number fields, enter 1 for single experiment time step and enter a list of column numbers (with each column number corresponding to a single timepoint) for animated time series. In the Color Scheme, “three color display with specified threshold” was selected and the specified threshold was entered in the specified threshold field. The other fields should be default selection. Then click the “Submit ”at the bottom of the Pathway Tools Omics Viewer webpage. When the *Oryza sativa Japonica* *group* metabolic map was generated, a metabolite can be identified by moving the mouse over the metabolite icon; the metabolite page or a related pathway page can be navigated by clicking on the metabolite icon [17]. 

### 4.6. Animation the Comparative Transcriptome Analysis Using Pathway Tools Omics Viewer

For single experiment time step, the metabolic map generated by Pathway Tools Omics Viewer is static but not animated. It is more ready and direct to visualize the expression patterns for single experiment time step if the metabolic map generated by Pathway Tools Omics Viewer is animated. However, in the Pathway Tools Omics Viewer, one comparative transcriptome analysis of interest, e.g., the differentially expressed genes between before and after infestation in the resistant rice plants, was considered as single experiment time step. Meanwhile, the single experiment time step can’t mimic animated time series by enter two 1 in the column number fields or copy the relative or absolute expression level of each differentially expressed gene in the second column for a third column in the validating of rice transcriptome data for Pathway Tools Omics Viewer process and enter 1 and 2 in the column number fields in the visualization of rice transcriptome data using Pathway Tools Omics Viewer process. To get animated pictures for comparative transcriptome analysis and more readily visualize the expression patterns, in this study, the individual metabolic pathways that were differentially regulated between before and after infestation in the resistant (R6_R0) and susceptible rice plants (S6_S0) respectively was taken as one combination for animated time series; while the individual metabolic pathways that were differentially regulated between resistant and susceptible rice plants before (R0_S0) and after infestation (R6_S6) respectively was taken as another combination for animated time series. Then mark the differentially regulated individual metabolic pathways between R6_S6 and R0_S0 or between R6_R0 and S6_S0 with green box. 

### 4.7. Identification of the Differentially Regulated Individual Metabolic Pathways by the Pathway Tools Omics Viewer

Although a pathway in the metabolic map generated by pathway tools can be navigated, in most cases, e.g., comparative transcriptome analysis of interest, it is more ideal and direct to get clear understanding of the differentially regulated metabolic pathways in more details if the differentially expressed genes and its differential expression values can be mapped to the individual metabolic pathway. Notably, the Pathway Tools Omics Viewer provides an alternative display to generate a table containing all individual metabolic pathways [17]. Therefore, to get the differentially regulated individual metabolic pathways with omics data painted onto the metabolic pathway, in the “Display Type”, the third option, “generate a table of individual pathways exceeding threshold”, was selected. Then do the others as do for visualization of rice transcriptomic data using Pathway Tools Omics Viewer as described above. According to the fold changes of the differentially expressed genes that belong to the corresponding individual metabolic pathways derived from the Pathway Tools Omics Viewer, the individual metabolic pathway was then classified into three groups, if all the differentially expressed genes were up-regulated or higher, the individual metabolic pathway was up-regulated or higher; if all the differentially expressed genes were down-regulated or lower, the individual metabolic pathway was down-regulated or lower; if the differentially expressed genes were both up-regulated and down-regulated or higher and lower, the individual metabolic pathway was both up-regulated and down-regulated or both higher and lower and marked up/down or higher/lower in this report.

### 4.8. Identification of Metabolic Pathways Potentially Related to SBPH Resistance by Pathway Tools Omics Viewer

To identify the most relevant metabolic pathways associated with SBPH resistance and susceptibility in rice, the web tool Venn (http://bioinfogp.cnb.csic.es/) was next employed. The differentially regulated individual metabolic pathways in the four comparisons that identified by the Pathway Tools Omics Viewer were represented in a Venn diagram. In the Venn diagram webpage, click the numbers in the Venn diagram to see the results and copy each result of 15 categories to a *microsoft* excel worksheet. The table in the *microsoft* excel worksheet contains five columns, the first row contains column header following metabolic pathway and four comparisons, one metabolic pathway per row. According to the result of the individual metabolic pathway that was classified into three groups mentioned as above, mark the individual metabolic pathway with up or down or up/down as well as higher or lower or higher/lower. By searching the metabolic pathway in the Gramene database or click the link of the metabolic pathway in the result that displayed as a table containing individual metabolic pathway, download the classification hierarchy for individual metabolic pathways from the rice pathway database (http://pathway.iplantcollaborative.org/).

### 4.9. Measurement of Free Amino Acids

The result of the identification of the metabolic pathway associated with resistance and susceptibility to SBPH showed that several kinds of the amino acids were correlated with resistance and susceptibility to SBPH, therefore, the concentrations of the free amino acids were then determined as described by Lei [62]. Briefly, leaf sheath power (0.2 g) was sampled, sulfosalicylic acid (3%) was then added to a volume of 4 mL for protein precipitation, and extracted in 80 °C water for 30 min. After cooling to room temperature, the extract was centrifuged at 13,500× *g* at 4 °C for 10 min. The extracted supernatant was then filtered through a 0.45-um membrane filter. Finally, the supernatant (100 µL) was determined by using the automatic amino acid analyzer L-8900 (Hitachi Construction Machinery Co., Ltd., Tokyo, Japan).

## Supplementary Materials

Supplementary materials can be found at http://www.mdpi.com/1422-0067/16/12/26128/s1.
